# Risk of death, thrombotic and hemorrhagic events in anticoagulated patients with atrial fibrillation and systemic autoimmune diseases: an analysis from a global federated dataset

**DOI:** 10.1007/s00392-024-02426-1

**Published:** 2024-03-06

**Authors:** Tommaso Bucci, Chiara Cardamone, Massimo Triggiani, Paul R. J. Ames, Gregory Y. H. Lip

**Affiliations:** 1 Liverpool Centre of Cardiovascular Science at University of Liverpool, Liverpool John Moores University and Liverpool Heart & Chest Hospital, Liverpool, UK; 2 Department of General and Specialized Surgery, Sapienza University of Rome, Rome, Italy; 3 Division of Allergy and Clinical Immunology, University of Salerno, Salerno, Italy; 4 Immune Response and Vascular Disease Unit, CEDOC, Nova University Lisbon, Rua Camara Pestana, Lisbon, Portugal; 5 Department of Haematology, Dumfries Royal Infirmary, Cargenbridge, Dumfries UK; 6 Danish Center for Health Services Research, Department of Clinical Medicine, Aalborg University, Aalborg, Denmark

**Keywords:** Atrial fibrillation, Autoimmunity, Cardiovascular events, Mortality, Oral anticoagulant

## Abstract

**Background:**

Growing evidence showing that systemic autoimmune diseases (SADs) are associated with a high risk of atrial fibrillation (AF). However, the impact of SAD on the clinical course of AF patients is largely unknown.

**Methods:**

Retrospective cohort study within a federated healthcare network (TriNetX). Using ICD codes, AF patients on anticoagulant therapy were categorized according to the presence of SAD (M32: Systemic Lupus Erythematosus (SLE); M33: Dermato-polymyositis (DMP); M34: Systemic Sclerosis (SSc); M35: Sjogren syndrome). The primary outcomes were the 5-year risks of (1) all-cause death, (2) thrombotic events (ischemic stroke, acute myocardial infarction, deep vein thrombosis, and pulmonary embolism), and (3) bleeding (intracranial (ICH) and gastrointestinal (GI)). Secondary outcomes were each component of the primary outcomes. Cox regression analysis after propensity score matching (PSM) was used to estimate hazard ratio (HR) and 95% confidence interval (95%CI).

**Results:**

We identified 16,098 AF patients with SAD (68.2 ± 13.4 years; 71.0% female) and 828,772 AF controls (70.7 ± 12.9 years, 41.1% females). After PSM, AF patients with SAD were associated with a higher risk of all-cause death (HR 1.13, 95%CI 1.09–1.71), thrombotic events (HR 1.37, 95%CI 1.32–1.43), and hemorrhagic events (HR 1.41, 95%CI 1.33–1.50) compared to AF controls without SAD. The highest risk of all-cause death and GI bleeding was associated with SSc, while the highest risk of thrombotic events and ICH was associated with SLE.

**Conclusion:**

AF patients with SAD are associated with a high risk of all-cause death, thrombotic, and hemorrhagic events. These patients merit careful follow-up and integrated care management to improve their prognosis.

**Graphical Abstract:**

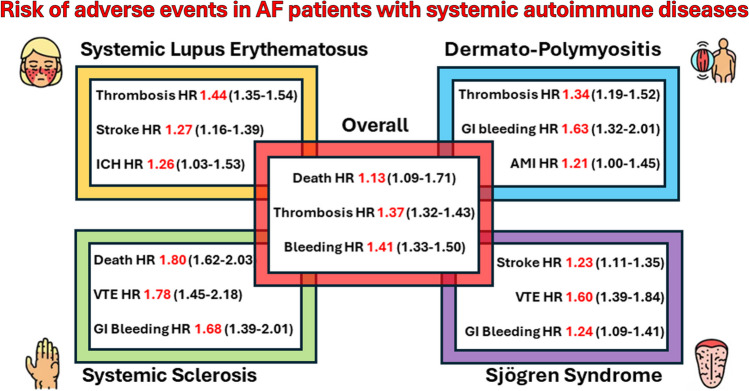

**Supplementary Information:**

The online version contains supplementary material available at 10.1007/s00392-024-02426-1.

## Introduction

Growing evidence shows that patients with systemic autoimmune diseases (SADs) have a high risk of incident atrial fibrillation (AF) [1, 2]. Indeed, the dysregulated inflammatory response that characterizes SAD can contribute to the electrical and structural left atrial remodeling mediated by the inflammasome activation, facilitating the onset and progression of AF [3, 4]. Moreover, SADs are often associated with several cardiovascular risk factors resulting from the multiorgan involvement or even the immunosuppressive treatments [5, 6]. These cardiovascular risk factors are main determinants of both the thromboembolic and hemorrhagic risks in AF patients [7, 8].

Although the number of studies reporting the association between SAD and AF has been rapidly increasing during the last few years, no study has specifically addressed the impact of SAD on the clinical course and outcomes of AF patients on anticoagulant therapy (OAC). The aim of this study was to evaluate the 5-year risk of adverse events in AF-SAD patients compared to AF patients without SAD.

## Methods

TriNetX is a research network utilized for several scientific purposes, compliant with the Health Insurance Portability and Accountability Act and the US federal law which protects the privacy and security of healthcare data, including de-identified data as per the de-identification standard of the HIPAA Privacy Rule (https://trinetx.com/real-world-resources/publications/). To gain access to the data in the TriNetX research network, requests are directed to TriNetX and a data sharing agreement is required. As a federated research network, studies using the TriNetX health research network do not need ethical approval as no patient identifiable identification is received.

### Study design

This was a retrospective observational study conducted within TriNetX, a global federated health research network with access to electronic medical records (EMRs) from academic and community hospitals covering approximately 80 million individuals, mainly located in the United States. Within this network, available data include demographics; healthcare utilization data (e.g., emergency department, inpatient, and outpatient attendance); diagnoses using International Classification of Diseases, Tenth Revision, Clinical Modification (ICD-10-CM) codes; laboratory results (Logical Observation Identifiers Names and Codes, LOINC); and medications (RxNorm/Veterans Affairs National Formulary (VANF) codes). More information can be found online (https://trinetx.com/company‐overview/).

### Cohort

The searches on the TriNetX online research platform were performed on 28 January 2024. Using ICD codes, AF patients (ICD-10-CM I48) on OAC (VANF code: BL110) were categorized into two groups: (1) AF-SAD patients (ICD-10-CM M32: Systemic Lupus Erythematosus (SLE); M33: Dermato-polymyositis (DPM); M34: Systemic Sclerosis (SSc); M35: Sjogren syndrome (SJs)) and (2) AF controls (without: M32–35: SAD or vasculitis, M04: autoinflammatory diseases, and M05–M14: Inflammatory arthropathies). More information about the codes utilized for building each population can be found on Supplementary Table 1. The searches were restricted to a specific time period comprised between 1 January 2000 and 31 December 2018. At the time of the search, 80 participating healthcare organizations had data available for individuals who met the study inclusion criteria. The baseline index event was the AF diagnosis reported in the TriNetX platform. Characteristics registered in the 1 year before the index event were considered the baseline characteristics. The clinical outcomes were identified via ICD-10-CM codes as follows: I63: ischemic stroke, G45: transient cerebral ischemic attack, I75: peripheral arterial thromboembolism, I21: acute myocardial infarction; I82.4: deep vein thrombosis of lower extremity; I26: pulmonary embolism; I60, I61, I62 for intracranial hemorrhage; and K92.1, K92.0, K92.2 for gastrointestinal (GI) bleeding (Supplementary Table 2). All-cause death was recorded using specific variable code within the TriNetX platform.

### Outcomes

The primary outcomes were the 5-year risk of (1) all-cause death; (2) a composite thrombotic outcome of ischemic stroke/transient cerebral ischemic attack/peripheral arterial thromboembolism, myocardial infarction, and deep vein thrombosis/pulmonary embolism; and (3) a composite hemorrhagic outcome of intracranial hemorrhage (ICH) and gastrointestinal (GI) bleeding. The secondary outcomes of interest were the 5-year risk of each component of the primary composite outcomes.

We performed a number of some sensitivity analyses to assess the robustness of our primary findings. First, we assessed the risk of primary and secondary outcomes in each SAD compared to AF controls. Second, we assessed the risks of primary outcomes in AF-SAD patients compared to AF controls, considering separately those treated with warfarin and non-vitamin K oral anticoagulants (NOACs). Thereafter, we directly compared AF-SAD patients on warfarin with those on NOAC.

### Statistical analyses

Baseline characteristics were compared using chi-squared tests for categorical variables and independent-sample *t*-tests for continuous variables. Propensity score matching (PSM) 1:1 with neighbor algorithm was used to control the differences in the comparison cohorts. Cohort matching was performed for age at index event, sex, ethnicity, arterial hypertension, diabetes, obesity, dyslipidemia, chronic kidney disease, ischemic heart disease, heart failure, previous cerebral infarction, and cardiovascular medications (β-blockers, antiarrhythmics (class Ia, class Ic, class III), diuretics, statins, antianginals, calcium channel blockers, angiotensin-converting enzyme inhibitors, angiotensin II receptor blockers, and platelet aggregation inhibitors). These variables were chosen because they may influence the risk of primary and secondary outcomes. Absolute standardized mean differences (ASDs) were used to show the distribution of demographic and clinical data among the groups and calculated as the difference in the means or proportions of a particular variable divided by the pooled estimate of standardized differences for that variable. Any baseline characteristic with an ASD < 0.100 was considered well matched. After PSM, Cox regression proportional hazard models were used to calculate hazard ratios (HRs) with 95% confidence intervals (CIs) for the risk of primary and secondary outcomes in AF-SAD patients compared to AF controls. Sensitivity analyses were performed as described above.

All tests were two-tailed and *p*-values of ≤ 0.05 were taken to indicate statistical significance. All analyses were performed in the TriNetX platform which incorporates R (v4.3.1, R Foundation for Statistical Computing, Vienna, Austria).

## Results

The initial cohorts consisted of 16,098 AF-SAD patients (68.2 ± 13.4 years, 71.0% females) and 828,772 AF controls without SAD (70.7 ± 12.9 years, 41.1% females). Before PSM, AF-SAD patients were younger, more commonly females, and Black African or Asian, and with a higher prevalence of obesity, hypertension, heart failure, diabetes, dyslipidemia, chronic kidney disease, ischemic heart disease, and previous stroke, compared to AF controls (Table 1).

**Table 1:** Baseline characteristics of patients with atrial fibrillation and autoimmune diseases before and after the propensity score matching

	Before propensity score match			After propensity score match		
	AF patients with SAD *n* = 16,098	AF controls *n* = 828,772	ASD	AF patients with SAD *n* = 15,686	AF controls *n* = 15,686	ASD
Age, years (± SD)	68.2 ± 13.4	70.7 ± 12.9	0.189	68.3 ± 13.3	68.2 ± 13.8	0.001
Female, *n* (%)	11,143 (71.0)	336,646 (41.1)	0.632	11,139 (71.0)	11,168 (71.2)	0.004
White	10,591 (67.5)	625,128 (76.3)	0.197	10,591 (67.5)	10,747 (68.5)	0.021
Black or African American	2037 (13.0)	57,341 (7.0)	0.201	2036 (13.0)	1985 (12.7)	0.010
Asian	864 (5.5)	17,914 (2.2)	0.173	861 (5.5)	770 (4.9)	0.045
Hypertension	9477 (60.4)	300,458 (36.7)	0.489	9473 (60.4)	9678 (61.7)	0.027
Obesity	2200 (14.0)	55,788 (6.8)	0.238	2198 (14.0)	2154 (13.7)	0.008
Diabetes mellitus	3843 (24.5)	124,227 (15.2)	0.236	3843 (24.5)	3963 (25.3)	0.018
Dyslipidemia	6152 (39.2)	204,038 (24.9)	0.310	6150 (39.2)	6282 (40.0)	0.017
Chronic kidney disease	3376 (21.5)	68,609 (8.4)	0.375	3372 (21.5)	3286 (20.9)	0.010
Ischemic heart disease	4756 (30.3)	159,938 (19.5)	0.251	4754 (30.3)	4821 (30.7)	0.009
Heart failure	4252 (27.1)	113,579 (13.9)	0.332	4249 (27.1)	4214 (26.9)	0.005
Cerebral infarction	1067 (6.8)	34,381 (4.2)	0.114	1067 (6.8)	1000 (6.4)	0.017
Antiarrhythmics	6042 (38.5)	195,395 (23.9)	0.320	6038 (38.5)	6127 (39.1)	0.012
Statins	5793 (36.9)	243,422 (29.7)	0.153	5792 (36.9)	5939 (37.9)	0.020
Beta blockers	7751 (49.4)	309,003 (37.7)	0.237	7747 (49.4)	7965 (50.8)	0.028
Diuretics	7158 (45.6)	235,578 (28.8)	0.354	7154 (45.6)	7146 (45.6)	0.001
Calcium channel blockers	5219 (33.3)	168,092 (20.5)	0.290	5215 (33.2)	5236 (33.4)	0.003
ACE inhibitors	3389 (21.6)	143,251 (17.5)	0.104	3387 (21.6)	3408 (21.7)	0.003
Angiotensin II inhibitors	2580 (16.4)	79,603 (9.7)	0.200	2578 (16.4)	2517 (16.0)	0.011
Platelet aggregation inhibitors	5599 (35.7)	211,950 (25.9)	0.214	5597 (35.7)	5653 (36.0)	0.007

*ACE* angiotensin-converting enzyme, *AF* atrial fibrillation, *SAD* systemic autoimmune disease, *SD* standard deviation, *ASD* absolute standardized mean difference

The number of primary outcomes recorded in AF-SAD patients and AF controls is reported in Table 2. Prior to PSM, AF-SAD patients were associated with a higher risk of all-cause death (HR 1.21, 95%CI 1.18–1.24), thrombotic (HR 1.67, 95%CI 1.63–1.72), and hemorrhagic events (HR 1.77, 95%CI 1.70–1.84) compared to AF controls (Table 2). With regard to the secondary outcomes, AF-SAD patients were at higher risk of stroke, myocardial infarction, deep vein thrombosis, pulmonary embolism, ICH, and GI bleeding compared to AF controls.

**Table 2:** 5-year risk of primary and secondary outcomes in patients with atrial fibrillation with systemic autoimmune disease

	Before PSM			After PSM		
	AF with systemic autoimmune disease *n* = 16,098	AF controls *n* = 828,772	HR (95%CI)	AF with systemic autoimmune disease *n* = 15,686	AF controls *n* = 15,686	HR (95%CI)
	Events *n* (%)	Events *n* (%)		Events *n* (%)	Events *n* (%)	
All-cause death	5506 (35.1)	223,696 (27.3)	1.21 (1.18–1.24)	5503 (35.1)	4704 (30.0)	1.13 (1.09–1.71)
Composite thrombotic outcome	5779 (36.8)	186,174 (22.7)	1.67 (1.63–1.72)	5778 (36.8)	4330 (27.6)	1.37 (1.32–1.43)
Stroke/transient ischemic attack/peripheral embolism	2776 (17.7)	96,480 (11.8)	1.46 (1.40–1.51)	2776 (17.7)	2177 (13.9)	1.25 (1.18–1.32)
Myocardial infarction	2158 (13.8)	71,612 (8.7)	1.50 (1.44–1.57)	2158 (13.8)	1765 (11.3)	1.18 (1.11–1.26)
Venous thromboembolism	2292 (14.6)	48,768 (5.9)	2.43 (2.33–2.53)	2291 (14.6)	1217 (7.8)	1.89 (1.76–2.03)
Composite hemorrhagic outcome	2682 (17.1)	76,643 (9.3)	1.77 (1.70–1.84)	2681 (17.1)	1864 (11.9)	1.41 (1.33–1.50)
Intracranial hemorrhage	597 (3.8)	22,491 (2.7)	1.30 (1.20–1.41)	597 (3.8)	513 (3.3)	1.12 (1.00–1.26)
Gastrointestinal bleeding	2221 (14.2)	56,803 (6.9)	1.97 (1.89–2.05)	2219 (14.1)	1360 (8.7)	1.53 (1.43–1.64)

*AF* atrial fibrillation, *PSM* propensity score matching, *HR* hazard ratio, *CI* confidence interval

**Fig. 1:**
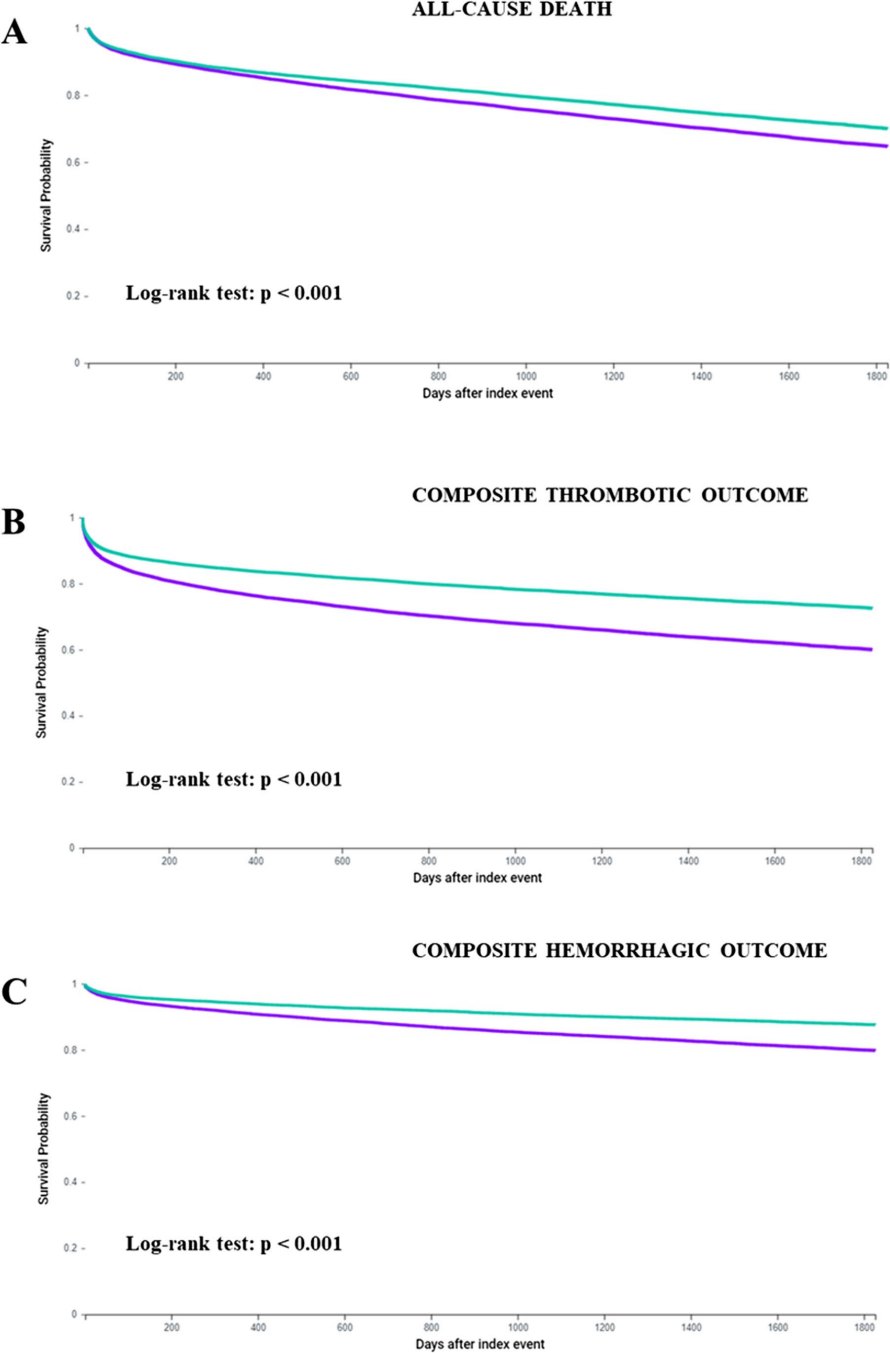
Survival curves for the primary outcomes in patients with atrial fibrillation and autoimmune diseases (purple) and control patients (green)

After PSM, 15,686 AF patients entered each group and no significative baseline differences were found between the two groups (Table 1). Consistent with the unmatched analysis, AF-SAD patients showed a significant higher risk of primary (Fig. 1, panels A, B, and C; Table 2) and secondary outcomes compared to AF controls (Table 2).

### Sensitivity analyses

In the first sensitivity analysis, after PSM, for the comparison with AF controls, we selected for each group (1) 5773 AF-SLE patients, (2) 1625 AF-DMP patients, (3) 1855 AF-SSc patients, and (4) 4694 AF-SJs patients (Supplementary Table 3–6). The number of events for each outcome in each SAD is reported in Table 3. The highest risk of all-cause death was associated with SSc (HR 1.80, 95%CI 1.62–2.03), while the highest risk of composite thrombotic events was associated with SLE (HR 1.44, 95%CI 1.35–1.54). Regarding the risk of composite hemorrhagic events, it was similar in SLE, DMP, and SSc but was the lowest in SJs (Table 3).

**Table 3:** 5-year risks of primary and secondary outcomes in each type of systemic autoimmune systemic autoimmune disease after propensity score matching

	Systemic lupus erythematosus *n* = 5773	Dermato-polymyositis *n* = 1625	Systemic sclerosis *n* = 1855	Sjogren syndrome *n* = 4694
	HR (95%CI)	HR (95%CI)	HR (95%CI)	HR (95%CI)
All-cause death	1.14 (1.07–1.22)	1.20 (1.06–1.35)	1.80 (1.62–2.03)	0.91 (0.85–1.01)
Composite thrombotic outcome	1.44 (1.35–1.54)	1.34 (1.19–1.52)	1.30 (1.15–1.47)	1.22 (1.14–1.32)
Stroke/transient ischemic attack/peripheral embolism	1.27 (1.16–1.39)	1.25 (1.05–1.49)	0.98 (0.82–1.18)	1.23 (1.11–1.35)
Myocardial infarction	1.27 (1.15–1.41)	1.21 (1.00–1.45)	1.24 (1.03–1.50)	1.02 (0.91–1.16)
Venous thromboembolism	1.91 (1.72–2.13)	1.87 (1.50–2.39)	1.78 (1.45–2.18)	1.60 (1.39–1.84)
Composite hemorrhagic outcome	1.51 (1.37–1.66)	1.54 (1.27–1.87)	1.52 (1.29–1.80)	1.14 (1.02–1.28)
Intracranial hemorrhage	1.26 (1.03–1.53)	1.30 (0.88–1.93)	0.99 (0.69–1.42)	0.92 (0.74–1.14)
Gastrointestinal bleeding	1.57 (1.41–1.75)	1.63 (1.32–2.01)	1.68 (1.39–2.01)	1.24 (1.09–1.41)

*HR* hazard ratio, *CI* confidence interval

Of the secondary outcomes, compared to AF controls, the risk of stroke was significantly higher in SLE, DMP, and SJs yet it was not significant in SSc (Table 3). The risk of myocardial infarction was increased in all SADs except for SSj, whereas the risk of DVT was increased in all AF-SAD patients (Table 3). With regard to hemorrhagic events, SLE was associated with the highest risk of ICH (HR 1.26, 95%CI 1.03–1.53), while the risk of GI bleeding was increased in all SADs (Table 3).

In the second sensitivity analysis, after PSM, we selected for each group 7611 patients on warfarin, 4800 patients on NOAC, and 4733 patients for the direct comparison between AF-SAD patients on warfarin with those on NOAC (Supplementary Table 7–9). In these analyses, the higher risk of primary and secondary outcomes in AF-SAD patients compared to AF controls was consistently independent of OAC type (Table 4). When directly compared, AF-SAD patients on warfarin showed a higher risk of all-cause death, and thrombotic events, and a non-significant trend for a higher risk of bleeding compared to those taking NOACs (Table 4). AF-SAD patients treated with warfarin were associated with a higher risk of deep vein thrombosis compared to those on NOAC, whereas a non-significant trend was found for arterial events, ICH, and GI bleeding (Table 4).

**Table 4:** 5-year risks of primary and secondary outcomes in patients with atrial fibrillation and autoimmune disease based on the oral anticoagulant type

	AF-SAD vs AF CTRL Warfarin	AF-SAD vs AF CTRL NOAC	AF-SAD on warfarin vs AF-SAD on NOAC
	HR (95%CI)	HR (95%CI)	HR (95%CI)
All-cause death	1.13 (1.07–1.20)	1.23 (1.14–1.37)	1.16 (1.08–1.25)
Composite thrombotic outcome	1.45 (1.38–1.53)	1.38 (1.28–1.49)	1.22 (1.14–1.30)
Stroke/transient ischemic attack/peripheral embolism	1.26 (1.17–1.36)	1.25 (1.13–1.16)	1.05 (0.96–1.16)
Myocardial infarction	1.28 (1.17–1.45)	1.24 (1.10–1.40)	1.02 (0.91–1.14)
Venous thromboembolism	1.83 (1.68–1.99)	1.89 (1.65–2.18)	1.53 (1.38–1.70)
Composite hemorrhagic outcome	1.35 (1.25–1.47)	1.59 (1.42–1.77)	1.09 (0.99–1.20)
Intracranial hemorrhage	1.13 (0.96–1.33)	1.16 (0.92–1.47)	1.18 (0.95–1.46)
Gastrointestinal bleeding	1.44 (1.32–1.57)	1.73 (1.53–1.96)	1.06 (0.96–1.80)

*AF* atrial fibrillation, *CTRL* controls, *NOAC* non-vitamin K anticoagulant, *HR* hazard ratio, *CI* confidence interval, *SAD* systemic autoimmune disease

## Discussion

In this study, our principal findings are as follows: (1) AF-SAD patients were associated with a higher risk of all-cause death, thrombotic, and hemorrhagic events compared to AF controls; (2) each different SAD is associated with a particular thrombotic and hemorrhagic risk profile; (3) the risk of adverse events in AF-SAD patients was independent of OAC type, although those taking warfarin had a higher risk of mortality and thrombotic events compared to those on NOAC.

In our study, AF-SAD patients had clinical phenotype characterized by younger age, and a high prevalence of female sex, Black African and Asian ethnicity, and cardiovascular risk factors. SAD predilects females and usually arises during the adolescence or young adulthood [9]. The high prevalence of certain ethnicities confirms previous epidemiological studies that have shown that the odds for SAD is higher, and the mean age of disease onset is lower, in Black Africans and Asians compared to Whites [10, 11]. The earlier onset of SAD implies a longer exposure to the inflammatory state that eventually favors an early AF onset with potential anticipation of cardiovascular events.

The higher risk of all-cause death in SAD patients, and particularly in those with SSc, has been previously reported in a retrospective study on 3,150,267 individuals, in which SAD was the leading cause of death among females in England and Wales [12], as well as another retrospective study on 711,247 individuals from the Netherlands, in which SAD was associated with a high mortality rate in females [13].

SAD patients are generally characterized by several risk factors that could increase the risk of death: immunodeficiency may favor the onset of infections or neoplasia; renal involvement can evolve in acute on chronic kidney disease; interstitial lung disease progressing to pulmonary fibrosis may induce respiratory failure, pulmonary hypertension, and heart failure, whereas the prolonged use of steroids can facilitate the onset of secondary Cushing syndrome, diabetes, dyslipidemia, and hypertension [14, 15]. Moreover, the proinflammatory state associated with SAD may heighten the risk of premature atherosclerosis that could lead to an increased risk of arterial events [6], and perturb Virchow’s triad including blood stasis, hypercoagulability, and endothelial injury, leading to an increased risk of venous thromboembolism [16].

Indeed, we found that AF-SAD patients had a higher risk of composite thrombotic outcomes, ischemic stroke, myocardial infarction, and deep venous thrombosis when compared to AF controls. This is in accord with a retrospective study on 98,308 adults with SAD and 198,044 controls enrolled from the MarketScan Commercial Claims databases, where SAD was associated with a sixfold increased risk for venous thromboembolism [17], and with another retrospective study on 136,120 hospitalized patients with SAD from the National Inpatient Sample in the United States, showing that patients with SAD had a higher risk of venous thromboembolism compared to controls [18]. Conversely, in the COMMAND VTE (COntemporary ManageMent AND outcomes in patients with Venous ThromboEmbolism) registry on 2332 patients with acute venous thromboembolism, the high risk for venous thromboembolism in SAD patients was related more to the use of corticosteroids than to the SAD itself [19].

Few studies have analyzed the overall risk of arterial events in SAD patients considering them as a unique clinical entity. A retrospective study on 216,291 hospitalized individuals from the Swedish Hospital Discharge Register showed that patients with SAD had a higher 1-year risk of stroke after discharge (HR 1.50, 95%CI 1.46–1.55) [20], whereas a retrospective study on 79,390 patients hospitalized for myocardial infarction from two Australian population-based datasets showed that SAD was associated with a higher 1-year risk of cardiovascular death (OR 1.71, 95%CI 1.51–1.94) [21].

We found that SLE was associated with the highest risk of composite thrombosis outcome. This has been extensively reported and the reasons are often but not always related to the presence of antiphospholipid antibodies [22]. The development of vasculitis and the enhanced atherosclerosis in SLE could provide other valid explanations [22]. In AF patients with DMP, we found the highest risk of myocardial infarction. In a case–control study on 774 patients with DMP from Canada, the risk of myocardial infarction was increased in DMP patients (HR 6.51, 95%CI 3.15–13.47) [23], whereas in a prospective study on 118 DMP patients followed for 6 years, a 16-fold increased risk of death from myocardial infarction was found [24]. SJs was associated with a high risk of stroke but the data about the risk of cardiovascular events in this disease are controversial. For example, one retrospective study on 4276 SJs patients obtained from the Registry of Catastrophic Illness in Taiwan found that SJs was not associated with a higher risk of stroke (OR 0.84, 95%CI 0.63–1.12), whereas a retrospective study on 102 well-characterized SJs patients showed a significant higher risk of cerebrovascular events (OR 3.83, 95%CI 1.27–11.5) [25].

Finally, we found a 51% higher risk of hemorrhagic events in AF-SAD patients. SSc was associated with the highest risk of GI bleeding, whereas SLE with the highest risk of ICH. This confirms the finding of two retrospective studies from Taiwan that showed a higher incidence of ICH in SLE patients (49.4 vs 10.2 per 100,000 person-year) [26], and a higher risk of GI bleeding in SSc patients (HR 3.93, 95%CI 2.52–6.13) [27]. The higher hemorrhagic risk in SSc was further confirmed by the data of the UK electronic primary care databases that showed a 21% increased risk of any bleeding (HR 1.21, 95%CI 1.00–1.54) [28]. These patients may develop autoantibodies against the coagulation factors VIII and IX leading to the acquired form of hemophilia A or B [29], or develop immune thrombocytopenia as a result of increased turnover or reduced production of platelets [30]. The presence of specific characteristics such as the GI mucosal abnormalities with fibrosis and small vessel vasculopathy in SSc patients or the presence of cerebral aneurysms in SLE patients could further increase this risk.

We noted that both the high thrombotic and the hemorrhagic risk in AF-SAD patients were independent of the OAC type and that patients prescribed NOAC showed a lower risk of all-cause death and thrombosis compared to those on warfarin. Indeed, NOACs are contraindicated in several conditions characterized by a high risk of thrombosis (e.g., antiphospholipid syndrome with triple positivity, advanced liver cirrhosis, end-stage renal disease) in which warfarin is still recommended, and this could have biased the results. Further prospective studies are needed to clarify the best antithrombotic strategies in this high-risk subgroup of AF patients.

The high risks associated with AF-SAD merit a more holistic care approach to managing these patients. Apart from stroke prevention and rhythm management, multidisciplinary cardiovascular preventive strategies, including comorbidity optimization and lifestyle modifications, are needed, aligned with current recommendations in guidelines, for an integrated care approach to AF management [31]. Indeed, adherence to the Atrial Fibrillation Better Care (ABC) pathway is associated with improved clinical outcomes in patients with AF [32, 33].

### Limitations

There are several limitations to acknowledge. First, this is a retrospective study and unmeasured bias could have influenced the results. Second, administrative data could fail to identify patients with AF or SAD, affecting the prognosis. Third, although we considered for the PSM the antiarrhythmics therapies, we cannot adjust for ablation or cardioversion procedures that occurred after the index event, for the possibility of introducing “immortal time biases,” thus making it impossible to have a comprehensive overview of those patients treated with rhythm control strategies. Fourth, the outcomes occurring outside the network may have not been well captured and could have influenced the risks associated with the presence or absence of SAD. Fifth, we did not analyze the risk of adverse event in each SAD based on the disease activity or severity, for the lack of these data. Lastly, we did not stratify the analyses based on the age, sex, ethnicity, steroids or immunosuppressive treatments, or the presence of social determinants of health.

## Conclusion

AF-SAD patients are associated with a high risk of all-cause death, thrombotic, and hemorrhagic events. These patients merit careful follow-up and integrated care management to improve their prognosis.

### Supplementary Information

Below is the link to the electronic supplementary material.

Supplementary file1 (DOCX 106 KB)

## Data Availability

The data underlying this article will be shared on reasonable request to the corresponding author.
