# Supplementary material for: Risk of death, thrombotic and hemorrhagic events in anticoagulated patients with atrial fibrillation and systemic autoimmune diseases: an analysis from a global federated dataset
Source: Clin Res Cardiol. 2024 Mar 6;113(6):942–50. doi: 10.1007/s00392-024-02426-1 (PMC11108877; doi:10.1007/s00392-024-02426-1)
Supplement: Supplementary file 1 — Supplementary file1 (DOCX 106 KB) [file 392_2024_2426_MOESM1_ESM.docx]

**Risk of death, thrombotic and hemorrhagic events in patients with atrial fibrillation and systemic autoimmune diseases.**

Tommaso Bucci, Chiara Cardamone, Massimo Triggiani, Paul R. J. Ames, Gregory Y.H. Lip

Supplementary material

TriNetX Database

The TriNetX data are collected from member healthcare organizations (HCO) and originates from their primary electronic health records (EHR) system. A typical HCO is a large academic health center with data coming from majority of its affiliates. A single HCO frequently has more than one facility, including main and satellite hospitals. The data are stored on the TriNetX database via a physical server at the institution’s data centre or a virtual hosted appliance. The TriNetX platform comprises of a series of these appliances connected into a federated network. This network can broadcast queries to each appliance. Results are subsequently collected and aggregated. Once the data are sent to the network, it is mapped to a standard and controlled set of clinical terminologies and undergoes a data quality assessment including ‘data cleaning’ that rejects records which do not meet the TriNetX quality standards. The TriNetX database performs internal and extensive data quality assessment with every refresh based on conformance, completeness, and plausibility (http://doi.org/10.13063/2327-9214.1244). HIPAA (Health Insurance Portability and Accountability Act) compliance of the clinical patient data is achieved using deidentification. Available data types within the network include demographics, diagnoses (represented by ICD-10-CM codes), procedures (coded in ICD-10-PCS or CPT), and measurements (coded to LOINC). While extensive information is provided about patients’ diagnoses and procedures, other variables (such as socioeconomic and lifetime factors are not comprehensively represented). The advantage of electronic health record data over insurance claim data is that both insured and uninsured patients are included. An advantage of electronic health record data over survey data is that the former represents the diagnostic rates in the population presenting to healthcare facilities. This provides an accurate account of the burden of specific diagnoses on healthcare systems. One primary limitation of relying on diagnoses is that they do not account for undiagnosed patients who might have a condition but have not yet received medical support. Another general limitation of electronic health record data is that a patient may be seen in different HCO for different components of their care. If one healthcare organization is not part of the federated network, then part of their medical records may not be available. Using a network of healthcare organizations, rather than a single site, limits this possibility but does not fully remove it. Propensity Score Matched Analyses Using logistic regression [Logistic Regression of the scikit-learn package in Python (version 3.7)], TriNetX performs a 1:1 greedy nearest neighbor matching model, with a caliper of 0.1 pooled standard deviations. To eliminate bias resulting from nearest neighbour algorithms, the orders of rows are randomized. Any baseline characteristic with a standardised mean difference between cohorts lower than 0.1 is deemed well matched (https://www.tandfonline.com/doi/full/10.1080/00273171.2011.568786).

**Supplementary Table 1. ICD-10-CM codes for inclusion and exclusion criteria in patients with atrial fibrillation.**

|  | Diagnosis, ICD-10-CM and VANF codes |
| --- | --- |
| **Patients with atrial fibrillation and autoimmune systemic disease** | |
| Inclusion criteria | - I48: Atrial fibrillation and flutter   and   - VANF code BL110: Anticoagulants   and   - M32: Systemic Lupus Erythematosus   or   - M33: Dermato-polymyositis   or   - M34: Systemic Sclerosis   or   - M35.0: Sjogren syndrome |
| Exclusion criteria | - none |
| **Patients with atrial fibrillation control** | |
| Inclusion criteria | - I48: Atrial fibrillation and flutter   and   - VANF code BL110: Anticoagulants |
| Exclusion criteria | - M30-M35: Systemic autoimmune disease   or   - M04: Autoinflammatory syndromes   or   - M05-M14: Inflammatory polyarthropathies |

**Supplementary Table 2. ICD-10-CM codes for the 5-year risk of all-cause death, thrombotic events, and bleeding.**

|  | **Diagnosis and ICD-10-CM code** |
| --- | --- |
| **All-cause death** | - Deceased (TriNetX variable) |
| **Composite thrombotic events** | - I63: Ischemic stroke - G45: Transient cerebral ischemic attack - I75: Peripheral arterial embolism - I21; Myocardial infarction - I82.4: Deep vein thrombosis of lower extremity - I26: Pulmonary embolism |
| **Bleeding** | 1. Intracranial hemorrhage:  - I61: Nontraumatic intracerebral hemorrhage - I60: Nontraumatic subarachnoid hemorrhage - I62: Other and unspecified nontraumatic intracranial hemorrhage  1. Gastrointestinal bleeding:  - K92.1: Melena - K92.2: Gastrointestinal hemorrhage, unspecified |

**Supplementary Table 3.** **Baseline characteristics of patients with atrial fibrillation and Systemic Lupus Erythematosus (Cohort 1) before and after the propensity score matching.**

| **Cohort 1 (N = 5,859) and cohort 2 (N = 828,772) characteristics before propensity score matching** | | | | | | | | | |
| --- | --- | --- | --- | --- | --- | --- | --- | --- | --- |
|  | **Demographics** | | | | | | | | |
|  |  | Cohort | |  | Mean ± SD | Patients | % of Cohort | P-Value | Std diff. |
|  |  | 1 2 | AI | Age at Index | 64.4 +/- 14.2 70.7 +/- 12.9 |  | 100% 100% | <0.001 | 0.467 |
|  |  | 1 2 | 2106-3 | White |  | 3,633 625,128 | 62.8% 76.3% | <0.001 | 0.297 |
|  |  | 1 2 | F | Female |  | 4,258 336,646 | 73.6% 41.1% | <0.001 | 0.696 |
|  |  | 1 2 | 2054-5 | Black or African American |  | 1,050 57,341 | 18.1% 7.0% | <0.001 | 0.341 |
|  |  | 1 2 | 2028-9 | Asian |  | 174 17,914 | 3.0% 2.2% | <0.001 | 0.052 |
|  | **Diagnosis** | | | | | | | | |
|  |  | Cohort | |  | Mean ± SD | Patients | % of Cohort | P-Value | Std diff. |
|  |  | 1 2 | I50 | Heart failure |  | 1,592 113,579 | 27.5% 13.9% | <0.001 | 0.342 |
|  |  | 1 2 | I20-I25 | Ischemic heart diseases |  | 1,730 159,938 | 29.9% 19.5% | <0.001 | 0.242 |
|  |  | 1 2 | I63 | Cerebral infarction |  | 409 34,381 | 7.1% 4.2% | <0.001 | 0.125 |
|  |  | 1 2 | E08-E13 | Diabetes mellitus |  | 1,359 124,227 | 23.5% 15.2% | <0.001 | 0.212 |
|  |  | 1 2 | E66 | Obesity |  | 896 55,788 | 15.5% 6.8% | <0.001 | 0.278 |
|  |  | 1 2 | E78 | Dyslipidemia |  | 2,113 204,038 | 36.5% 24.9% | <0.001 | 0.254 |
|  |  | 1 2 | N18 | Chronic kidney disease |  | 1,486 68,609 | 25.7% 8.4% | <0.001 | 0.473 |
|  |  | 1 2 | I10-I1A | Hypertension |  | 3,489 300,458 | 60.3% 36.7% | <0.001 | 0.486 |
|  | **Medication** | | | | | | | | |
|  |  | Cohort | |  | Mean ± SD | Patients | % of Cohort | P-Value | Std diff. |
|  |  | 1 2 | CV350 | ANTILIPEMIC AGENTS |  | 2,054 243,422 | 35.5% 29.7% | <0.001 | 0.124 |
|  |  | 1 2 | CV700 | DIURETICS |  | 2,510 235,578 | 43.4% 28.8% | <0.001 | 0.308 |
|  |  | 1 2 | CV300 | ANTIARRHYTHMICS |  | 2,210 195,395 | 38.2% 23.9% | <0.001 | 0.314 |
|  |  | 1 2 | CV100 | BETA BLOCKERS/RELATED |  | 2,896 309,003 | 50.1% 37.7% | <0.001 | 0.250 |
|  |  | 1 2 | CV200 | CALCIUM CHANNEL BLOCKERS |  | 1,868 168,092 | 32.3% 20.5% | <0.001 | 0.269 |
|  |  | 1 2 | CV800 | ACE INHIBITORS |  | 1,200 143,251 | 20.7% 17.5% | <0.001 | 0.083 |
|  |  | 1 2 | CV805 | ANGIOTENSIN II RECEPTOR INHIBITOR |  | 887 79,603 | 15.3% 9.7% | <0.001 | 0.170 |
|  |  | 1 2 | BL117 | PLATELET AGGREGATION INHIBITORS |  | 2,035 211,950 | 35.2% 25.9% | <0.001 | 0.203 |
| **Cohort 1 (N = 5,773) and cohort 2 (N = 5,773) characteristics after propensity score matching** | | | | | | | | | |
|  | **Demographics** | | | | | | | | |
|  |  | Cohort | |  | Mean ± SD | Patients | % of Cohort | P-Value | Std diff. |
|  |  | 1 2 | AI | Age at Index | 64.5 +/- 14.1 64.2 +/- 15.5 |  | 100% 100% | 0.339 | 0.018 |
|  |  | 1 2 | 2106-3 | White |  | 3,633 3,722 | 62.9% 64.5% | 0.085 | 0.032 |
|  |  | 1 2 | F | Female |  | 4,245 4,230 | 73.5% 73.3% | 0.752 | 0.006 |
|  |  | 1 2 | 2054-5 | Black or African American |  | 1,038 1,055 | 18.0% 18.3% | 0.681 | 0.008 |
|  |  | 1 2 | 2028-9 | Asian |  | 173 154 | 3.0% 2.7% | 0.286 | 0.020 |
|  | **Diagnosis** | | | | | | | | |
|  |  | Cohort | |  | Mean ± SD | Patients | % of Cohort | P-Value | Std diff. |
|  |  | 1 2 | I50 | Heart failure |  | 1,581 1,588 | 27.4% 27.5% | 0.884 | 0.003 |
|  |  | 1 2 | I20-I25 | Ischemic heart diseases |  | 1,721 1,747 | 29.8% 30.3% | 0.598 | 0.010 |
|  |  | 1 2 | I63 | Cerebral infarction |  | 408 389 | 7.1% 6.7% | 0.485 | 0.013 |
|  |  | 1 2 | E08-E13 | Diabetes mellitus |  | 1,356 1,327 | 23.5% 23.0% | 0.523 | 0.012 |
|  |  | 1 2 | E66 | Obesity |  | 889 894 | 15.4% 15.5% | 0.898 | 0.002 |
|  |  | 1 2 | E78 | Dyslipidemia |  | 2,108 2,116 | 36.5% 36.7% | 0.877 | 0.003 |
|  |  | 1 2 | N18 | Chronic kidney disease |  | 1,473 1,436 | 25.5% 24.9% | 0.428 | 0.015 |
|  |  | 1 2 | I10-I1A | Hypertension |  | 3,476 3,476 | 60.2% 60.2% | 1 | <0.001 |
|  | **Medication** | | | | | | | | |
|  |  | Cohort | |  | Mean ± SD | Patients | % of Cohort | P-Value | Std diff. |
|  |  | 1 2 | CV350 | ANTILIPEMIC AGENTS |  | 2,052 2,077 | 35.5% 36.0% | 0.627 | 0.009 |
|  |  | 1 2 | CV700 | DIURETICS |  | 2,499 2,507 | 43.3% 43.4% | 0.881 | 0.003 |
|  |  | 1 2 | CV300 | ANTIARRHYTHMICS |  | 2,198 2,219 | 38.1% 38.4% | 0.688 | 0.007 |
|  |  | 1 2 | CV100 | BETA BLOCKERS/RELATED |  | 2,884 2,888 | 50.0% 50.0% | 0.941 | 0.001 |
|  |  | 1 2 | CV200 | CALCIUM CHANNEL BLOCKERS |  | 1,858 1,881 | 32.2% 32.6% | 0.647 | 0.009 |
|  |  | 1 2 | CV800 | ACE INHIBITORS |  | 1,193 1,213 | 20.7% 21.0% | 0.647 | 0.009 |
|  |  | 1 2 | CV805 | ANGIOTENSIN II RECEPTOR INHIBITOR |  | 883 871 | 15.3% 15.1% | 0.756 | 0.006 |
|  |  | 1 2 | BL117 | PLATELET AGGREGATION INHIBITORS |  | 2,028 2,021 | 35.1% 35.0% | 0.891 | 0.003 |

**Supplementary table 4.** **Baseline characteristics of patients with atrial fibrillation and Dermato-Polymyositis (Cohort 1) before and after the propensity score matching.**

| **Cohort 1 (N = 1,658) and cohort 2 (N = 828,772) characteristics before propensity score matching** | | | | | | | | | |
| --- | --- | --- | --- | --- | --- | --- | --- | --- | --- |
|  | **Demographics** | | | | | | | | |
|  |  | Cohort | |  | Mean ± SD | Patients | % of Cohort | P-Value | Std diff. |
|  |  | 1 2 | AI | Age at Index | 69.1 +/- 12.4 70.7 +/- 12.9 |  | 100% 100% | <0.001 | 0.128 |
|  |  | 1 2 | 2106-3 | White |  | 1,131 625,128 | 69.6% 76.3% | <0.001 | 0.151 |
|  |  | 1 2 | F | Female |  | 820 336,646 | 50.5% 41.1% | <0.001 | 0.189 |
|  |  | 1 2 | 2054-5 | Black or African American |  | 234 57,341 | 14.4% 7.0% | <0.001 | 0.241 |
|  |  | 1 2 | 2028-9 | Asian |  | 69 17,914 | 4.2% 2.2% | <0.001 | 0.117 |
|  | **Diagnosis** | | | | | | | | |
|  |  | Cohort | |  | Mean ± SD | Patients | % of Cohort | P-Value | Std diff. |
|  |  | 1 2 | I50 | Heart failure |  | 472 113,579 | 29.0% 13.9% | <0.001 | 0.376 |
|  |  | 1 2 | I20-I25 | Ischemic heart diseases |  | 550 159,938 | 33.8% 19.5% | <0.001 | 0.328 |
|  |  | 1 2 | I63 | Cerebral infarction |  | 106 34,381 | 6.5% 4.2% | <0.001 | 0.103 |
|  |  | 1 2 | E08-E13 | Diabetes mellitus |  | 656 124,227 | 40.4% 15.2% | <0.001 | 0.587 |
|  |  | 1 2 | E66 | Obesity |  | 264 55,788 | 16.2% 6.8% | <0.001 | 0.299 |
|  |  | 1 2 | E78 | Dyslipidemia |  | 720 204,038 | 44.3% 24.9% | <0.001 | 0.417 |
|  |  | 1 2 | N18 | Chronic kidney disease |  | 322 68,609 | 19.8% 8.4% | <0.001 | 0.333 |
|  |  | 1 2 | I10-I1A | Hypertension |  | 1,007 300,458 | 62.0% 36.7% | <0.001 | 0.523 |
|  | **Medication** | | | | | | | | |
|  |  | Cohort | |  | Mean ± SD | Patients | % of Cohort | P-Value | Std diff. |
|  |  | 1 2 | CV350 | ANTILIPEMIC AGENTS |  | 652 243,422 | 40.1% 29.7% | <0.001 | 0.220 |
|  |  | 1 2 | CV700 | DIURETICS |  | 773 235,578 | 47.6% 28.8% | <0.001 | 0.395 |
|  |  | 1 2 | CV300 | ANTIARRHYTHMICS |  | 598 195,395 | 36.8% 23.9% | <0.001 | 0.285 |
|  |  | 1 2 | CV100 | BETA BLOCKERS/RELATED |  | 851 309,003 | 52.4% 37.7% | <0.001 | 0.298 |
|  |  | 1 2 | CV200 | CALCIUM CHANNEL BLOCKERS |  | 498 168,092 | 30.6% 20.5% | <0.001 | 0.234 |
|  |  | 1 2 | CV800 | ACE INHIBITORS |  | 455 143,251 | 28.0% 17.5% | <0.001 | 0.253 |
|  |  | 1 2 | CV805 | ANGIOTENSIN II RECEPTOR INHIBITOR |  | 254 79,603 | 15.6% 9.7% | <0.001 | 0.178 |
|  |  | 1 2 | BL117 | PLATELET AGGREGATION INHIBITORS |  | 606 211,950 | 37.3% 25.9% | <0.001 | 0.248 |
| **Cohort 1 (N = 1,625) and cohort 2 (N = 1,625) characteristics after propensity score matching** | | | | | | | | | |
|  | **Demographics** | | | | | | | | |
|  |  | Cohort | |  | Mean ± SD | Patients | % of Cohort | P-Value | Std diff. |
|  |  | 1 2 | AI | Age at Index | 69.1 +/- 12.4 68.8 +/- 13.4 |  | 100% 100% | 0.447 | 0.027 |
|  |  | 1 2 | 2106-3 | White |  | 1,131 1,092 | 69.6% 67.2% | 0.141 | 0.052 |
|  |  | 1 2 | F | Female |  | 820 857 | 50.5% 52.7% | 0.194 | 0.046 |
|  |  | 1 2 | 2054-5 | Black or African American |  | 234 245 | 14.4% 15.1% | 0.586 | 0.019 |
|  |  | 1 2 | 2028-9 | Asian |  | 69 68 | 4.2% 4.2% | 0.930 | 0.003 |
|  | **Diagnosis** | | | | | | | | |
|  |  | Cohort | |  | Mean ± SD | Patients | % of Cohort | P-Value | Std diff. |
|  |  | 1 2 | I50 | Heart failure |  | 472 471 | 29.0% 29.0% | 0.969 | 0.001 |
|  |  | 1 2 | I20-I25 | Ischemic heart diseases |  | 550 579 | 33.8% 35.6% | 0.285 | 0.037 |
|  |  | 1 2 | I63 | Cerebral infarction |  | 106 100 | 6.5% 6.2% | 0.666 | 0.015 |
|  |  | 1 2 | E08-E13 | Diabetes mellitus |  | 656 631 | 40.4% 38.8% | 0.370 | 0.031 |
|  |  | 1 2 | E66 | Obesity |  | 264 263 | 16.2% 16.2% | 0.962 | 0.002 |
|  |  | 1 2 | E78 | Dyslipidemia |  | 720 713 | 44.3% 43.9% | 0.805 | 0.009 |
|  |  | 1 2 | N18 | Chronic kidney disease |  | 322 313 | 19.8% 19.3% | 0.691 | 0.014 |
|  |  | 1 2 | I10-I1A | Hypertension |  | 1,007 1,014 | 62.0% 62.4% | 0.800 | 0.009 |
|  | **Medication** | | | | | | | | |
|  |  | Cohort | |  | Mean ± SD | Patients | % of Cohort | P-Value | Std diff. |
|  |  | 1 2 | CV350 | ANTILIPEMIC AGENTS |  | 652 669 | 40.1% 41.2% | 0.544 | 0.021 |
|  |  | 1 2 | CV700 | DIURETICS |  | 773 762 | 47.6% 46.9% | 0.699 | 0.014 |
|  |  | 1 2 | CV300 | ANTIARRHYTHMICS |  | 598 629 | 36.8% 38.7% | 0.262 | 0.039 |
|  |  | 1 2 | CV100 | BETA BLOCKERS/RELATED |  | 851 842 | 52.4% 51.8% | 0.752 | 0.011 |
|  |  | 1 2 | CV200 | CALCIUM CHANNEL BLOCKERS |  | 498 495 | 30.6% 30.5% | 0.909 | 0.004 |
|  |  | 1 2 | CV800 | ACE INHIBITORS |  | 455 459 | 28.0% 28.2% | 0.876 | 0.005 |
|  |  | 1 2 | CV805 | ANGIOTENSIN II RECEPTOR INHIBITOR |  | 254 251 | 15.6% 15.4% | 0.885 | 0.005 |
|  |  | 1 2 | BL117 | PLATELET AGGREGATION INHIBITORS |  | 606 631 | 37.3% 38.8% | 0.366 | 0.032 |

**Supplementary Table 5. Baseline characteristics of patients with atrial fibrillation and Systemic Sclerosis (Cohort 1) before and after the propensity score matching.**

| **Cohort 1 (N = 1,891) and cohort 2 (N = 828,772) characteristics before propensity score matching** | | | | | | | | | |
| --- | --- | --- | --- | --- | --- | --- | --- | --- | --- |
|  | **Demographics** | | | | | | | | |
|  |  | Cohort | |  | Mean ± SD | Patients | % of Cohort | P-Value | Std diff. |
|  |  | 1 2 | AI | Age at Index | 68.2 +/- 12.0 70.7 +/- 12.9 |  | 100% 100% | <0.001 | 0.201 |
|  |  | 1 2 | 2106-3 | White |  | 1,294 625,128 | 69.8% 76.3% | <0.001 | 0.148 |
|  |  | 1 2 | F | Female |  | 1,266 336,646 | 68.2% 41.1% | <0.001 | 0.567 |
|  |  | 1 2 | 2054-5 | Black or African American |  | 246 57,341 | 13.3% 7.0% | <0.001 | 0.209 |
|  |  | 1 2 | 2028-9 | Asian |  | 55 17,914 | 3.0% 2.2% | 0.022 | 0.049 |
|  | **Diagnosis** | | | | | | | | |
|  |  | Cohort | |  | Mean ± SD | Patients | % of Cohort | P-Value | Std diff. |
|  |  | 1 2 | I50 | Heart failure |  | 620 113,579 | 33.4% 13.9% | <0.001 | 0.473 |
|  |  | 1 2 | I20-I25 | Ischemic heart diseases |  | 583 159,938 | 31.4% 19.5% | <0.001 | 0.276 |
|  |  | 1 2 | I63 | Cerebral infarction |  | 86 34,381 | 4.6% 4.2% | 0.346 | 0.021 |
|  |  | 1 2 | E08-E13 | Diabetes mellitus |  | 393 124,227 | 21.2% 15.2% | <0.001 | 0.157 |
|  |  | 1 2 | E66 | Obesity |  | 220 55,788 | 11.9% 6.8% | <0.001 | 0.174 |
|  |  | 1 2 | E78 | Dyslipidemia |  | 635 204,038 | 34.2% 24.9% | <0.001 | 0.205 |
|  |  | 1 2 | N18 | Chronic kidney disease |  | 385 68,609 | 20.8% 8.4% | <0.001 | 0.356 |
|  |  | 1 2 | I10-I1A | Hypertension |  | 1,025 300,458 | 55.3% 36.7% | <0.001 | 0.379 |
|  | **Medication** | | | | | | | | |
|  |  | Cohort | |  | Mean ± SD | Patients | % of Cohort | P-Value | Std diff. |
|  |  | 1 2 | CV350 | ANTILIPEMIC AGENTS |  | 649 243,422 | 35.0% 29.7% | <0.001 | 0.113 |
|  |  | 1 2 | CV700 | DIURETICS |  | 938 235,578 | 50.6% 28.8% | <0.001 | 0.457 |
|  |  | 1 2 | CV300 | ANTIARRHYTHMICS |  | 707 195,395 | 38.1% 23.9% | <0.001 | 0.312 |
|  |  | 1 2 | CV100 | BETA BLOCKERS/RELATED |  | 824 309,003 | 44.4% 37.7% | <0.001 | 0.137 |
|  |  | 1 2 | CV200 | CALCIUM CHANNEL BLOCKERS |  | 675 168,092 | 36.4% 20.5% | <0.001 | 0.357 |
|  |  | 1 2 | CV800 | ACE INHIBITORS |  | 367 143,251 | 19.8% 17.5% | 0.009 | 0.059 |
|  |  | 1 2 | CV805 | ANGIOTENSIN II RECEPTOR INHIBITOR |  | 227 79,603 | 12.2% 9.7% | <0.001 | 0.081 |
|  |  | 1 2 | BL117 | PLATELET AGGREGATION INHIBITORS |  | 627 211,950 | 33.8% 25.9% | <0.001 | 0.174 |
| **Cohort 1 (N = 1,855) and cohort 2 (N = 1,855) characteristics after propensity score matching** | | | | | | | | | |
|  | **Demographics** | | | | | | | | |
|  |  | Cohort | |  | Mean ± SD | Patients | % of Cohort | P-Value | Std diff. |
|  |  | 1 2 | AI | Age at Index | 68.2 +/- 12.0 68.1 +/- 13.6 |  | 100% 100% | 0.823 | 0.007 |
|  |  | 1 2 | 2106-3 | White |  | 1,294 1,270 | 69.8% 68.5% | 0.394 | 0.028 |
|  |  | 1 2 | F | Female |  | 1,266 1,257 | 68.2% 67.8% | 0.751 | 0.010 |
|  |  | 1 2 | 2054-5 | Black or African American |  | 246 259 | 13.3% 14.0% | 0.534 | 0.020 |
|  |  | 1 2 | 2028-9 | Asian |  | 55 57 | 3.0% 3.1% | 0.848 | 0.006 |
|  | **Diagnosis** | | | | | | | | |
|  |  | Cohort | |  | Mean ± SD | Patients | % of Cohort | P-Value | Std diff. |
|  |  | 1 2 | I50 | Heart failure |  | 620 624 | 33.4% 33.6% | 0.889 | 0.005 |
|  |  | 1 2 | I20-I25 | Ischemic heart diseases |  | 583 587 | 31.4% 31.6% | 0.888 | 0.005 |
|  |  | 1 2 | I63 | Cerebral infarction |  | 86 84 | 4.6% 4.5% | 0.875 | 0.005 |
|  |  | 1 2 | E08-E13 | Diabetes mellitus |  | 393 405 | 21.2% 21.8% | 0.632 | 0.016 |
|  |  | 1 2 | E66 | Obesity |  | 220 212 | 11.9% 11.4% | 0.682 | 0.013 |
|  |  | 1 2 | E78 | Dyslipidemia |  | 635 662 | 34.2% 35.7% | 0.353 | 0.031 |
|  |  | 1 2 | N18 | Chronic kidney disease |  | 385 382 | 20.8% 20.6% | 0.903 | 0.004 |
|  |  | 1 2 | I10-I1A | Hypertension |  | 1,025 1,044 | 55.3% 56.3% | 0.530 | 0.021 |
|  | **Medication** | | | | | | | | |
|  |  | Cohort | |  | Mean ± SD | Patients | % of Cohort | P-Value | Std diff. |
|  |  | 1 2 | CV350 | ANTILIPEMIC AGENTS |  | 649 672 | 35.0% 36.2% | 0.430 | 0.026 |
|  |  | 1 2 | CV700 | DIURETICS |  | 938 945 | 50.6% 50.9% | 0.818 | 0.008 |
|  |  | 1 2 | CV300 | ANTIARRHYTHMICS |  | 707 716 | 38.1% 38.6% | 0.761 | 0.010 |
|  |  | 1 2 | CV100 | BETA BLOCKERS/RELATED |  | 824 833 | 44.4% 44.9% | 0.766 | 0.010 |
|  |  | 1 2 | CV200 | CALCIUM CHANNEL BLOCKERS |  | 675 669 | 36.4% 36.1% | 0.838 | 0.007 |
|  |  | 1 2 | CV800 | ACE INHIBITORS |  | 367 383 | 19.8% 20.6% | 0.513 | 0.021 |
|  |  | 1 2 | CV805 | ANGIOTENSIN II RECEPTOR INHIBITOR |  | 227 240 | 12.2% 12.9% | 0.520 | 0.021 |
|  |  | 1 2 | BL117 | PLATELET AGGREGATION INHIBITORS |  | 627 611 | 33.8% 32.9% | 0.577 | 0.018 |

**Supplementary table 6. Supplementary Table 5. Baseline characteristics of patients with atrial fibrillation and Sjogren Syndrome (Cohort 1) before and after the propensity score matching.**

| **Cohort 1 (N = 4,919) and cohort 2 (N = 828,772) characteristics before propensity score matching** | | | | | | | | | |
| --- | --- | --- | --- | --- | --- | --- | --- | --- | --- |
|  | **Demographics** | | | | | | | | |
|  |  | Cohort | |  | Mean ± SD | Patients | % of Cohort | P-Value | Std diff. |
|  |  | 1 2 | AI | Age at Index | 74.1 +/- 10.8 70.7 +/- 12.9 |  | 100% 100% | <0.001 | 0.285 |
|  |  | 1 2 | 2106-3 | White |  | 3,464 625,128 | 73.8% 76.3% | <0.001 | 0.058 |
|  |  | 1 2 | F | Female |  | 3,378 336,646 | 72.0% 41.1% | <0.001 | 0.655 |
|  |  | 1 2 | 2054-5 | Black or African American |  | 239 57,341 | 5.1% 7.0% | <0.001 | 0.080 |
|  |  | 1 2 | 2028-9 | Asian |  | 403 17,914 | 8.6% 2.2% | <0.001 | 0.286 |
|  | **Diagnosis** | | | | | | | | |
|  |  | Cohort | |  | Mean ± SD | Patients | % of Cohort | P-Value | Std diff. |
|  |  | 1 2 | I50 | Heart failure |  | 1,073 113,579 | 22.9% 13.9% | <0.001 | 0.234 |
|  |  | 1 2 | I20-I25 | Ischemic heart diseases |  | 1,402 159,938 | 29.9% 19.5% | <0.001 | 0.242 |
|  |  | 1 2 | I63 | Cerebral infarction |  | 357 34,381 | 7.6% 4.2% | <0.001 | 0.145 |
|  |  | 1 2 | E08-E13 | Diabetes mellitus |  | 1,117 124,227 | 23.8% 15.2% | <0.001 | 0.219 |
|  |  | 1 2 | E66 | Obesity |  | 589 55,788 | 12.5% 6.8% | <0.001 | 0.195 |
|  |  | 1 2 | E78 | Dyslipidemia |  | 2,136 204,038 | 45.5% 24.9% | <0.001 | 0.442 |
|  |  | 1 2 | N18 | Chronic kidney disease |  | 816 68,609 | 17.4% 8.4% | <0.001 | 0.271 |
|  |  | 1 2 | I10-I1A | Hypertension |  | 2,938 300,458 | 62.6% 36.7% | <0.001 | 0.537 |
|  | **Medication** | | | | | | | | |
|  |  | Cohort | |  | Mean ± SD | Patients | % of Cohort | P-Value | Std diff. |
|  |  | 1 2 | CV350 | ANTILIPEMIC AGENTS |  | 1,908 243,422 | 40.6% 29.7% | <0.001 | 0.230 |
|  |  | 1 2 | CV700 | DIURETICS |  | 2,095 235,578 | 44.6% 28.8% | <0.001 | 0.334 |
|  |  | 1 2 | CV300 | ANTIARRHYTHMICS |  | 1,820 195,395 | 38.8% 23.9% | <0.001 | 0.326 |
|  |  | 1 2 | CV100 | BETA BLOCKERS/RELATED |  | 2,356 309,003 | 50.2% 37.7% | <0.001 | 0.253 |
|  |  | 1 2 | CV200 | CALCIUM CHANNEL BLOCKERS |  | 1,534 168,092 | 32.7% 20.5% | <0.001 | 0.278 |
|  |  | 1 2 | CV800 | ACE INHIBITORS |  | 958 143,251 | 20.4% 17.5% | <0.001 | 0.075 |
|  |  | 1 2 | CV805 | ANGIOTENSIN II RECEPTOR INHIBITOR |  | 952 79,603 | 20.3% 9.7% | <0.001 | 0.299 |
|  |  | 1 2 | BL117 | PLATELET AGGREGATION INHIBITORS |  | 1,675 211,950 | 35.7% 25.9% | <0.001 | 0.214 |
| **Cohort 1 (N = 4,694) and cohort 2 (N = 4,694) characteristics after propensity score matching** | | | | | | | | | |
|  | **Demographics** | | | | | | | | |
|  |  | Cohort | |  | Mean ± SD | Patients | % of Cohort | P-Value | Std diff. |
|  |  | 1 2 | AI | Age at Index | 74.1 +/- 10.8 73.8 +/- 11.3 |  | 100% 100% | 0.198 | 0.027 |
|  |  | 1 2 | 2106-3 | White |  | 3,464 3,536 | 73.8% 75.3% | 0.088 | 0.035 |
|  |  | 1 2 | F | Female |  | 3,378 3,400 | 72.0% 72.4% | 0.612 | 0.010 |
|  |  | 1 2 | 2054-5 | Black or African American |  | 239 240 | 5.1% 5.1% | 0.963 | 0.001 |
|  |  | 1 2 | 2028-9 | Asian |  | 403 322 | 8.6% 6.9% | 0.002 | 0.065 |
|  | **Diagnosis** | | | | | | | | |
|  |  | Cohort | |  | Mean ± SD | Patients | % of Cohort | P-Value | Std diff. |
|  |  | 1 2 | I50 | Heart failure |  | 1,073 1,105 | 22.9% 23.5% | 0.434 | 0.016 |
|  |  | 1 2 | I20-I25 | Ischemic heart diseases |  | 1,402 1,440 | 29.9% 30.7% | 0.393 | 0.018 |
|  |  | 1 2 | I63 | Cerebral infarction |  | 357 400 | 7.6% 8.5% | 0.103 | 0.034 |
|  |  | 1 2 | E08-E13 | Diabetes mellitus |  | 1,117 1,136 | 23.8% 24.2% | 0.646 | 0.009 |
|  |  | 1 2 | E66 | Obesity |  | 589 555 | 12.5% 11.8% | 0.283 | 0.022 |
|  |  | 1 2 | E78 | Dyslipidemia |  | 2,136 2,178 | 45.5% 46.4% | 0.384 | 0.018 |
|  |  | 1 2 | N18 | Chronic kidney disease |  | 816 816 | 17.4% 17.4% | 1 | <0.001 |
|  |  | 1 2 | I10-I1A | Hypertension |  | 2,938 3,004 | 62.6% 64.0% | 0.158 | 0.029 |
|  | **Medication** | | | | | | | | |
|  |  | Cohort | |  | Mean ± SD | Patients | % of Cohort | P-Value | Std diff. |
|  |  | 1 2 | CV350 | ANTILIPEMIC AGENTS |  | 1,908 2,021 | 40.6% 43.1% | 0.018 | 0.049 |
|  |  | 1 2 | CV700 | DIURETICS |  | 2,095 2,160 | 44.6% 46.0% | 0.178 | 0.028 |
|  |  | 1 2 | CV300 | ANTIARRHYTHMICS |  | 1,820 1,949 | 38.8% 41.5% | 0.007 | 0.056 |
|  |  | 1 2 | CV100 | BETA BLOCKERS/RELATED |  | 2,356 2,465 | 50.2% 52.5% | 0.024 | 0.046 |
|  |  | 1 2 | CV200 | CALCIUM CHANNEL BLOCKERS |  | 1,534 1,543 | 32.7% 32.9% | 0.843 | 0.004 |
|  |  | 1 2 | CV800 | ACE INHIBITORS |  | 958 996 | 20.4% 21.2% | 0.334 | 0.020 |
|  |  | 1 2 | CV805 | ANGIOTENSIN II RECEPTOR INHIBITOR |  | 952 917 | 20.3% 19.5% | 0.366 | 0.019 |
|  |  | 1 2 | BL117 | PLATELET AGGREGATION INHIBITORS |  | 1,675 1,762 | 35.7% 37.5% | 0.062 | 0.038 |

**Supplementary table 7. Baseline characteristics of patients with atrial fibrillation and autoimmune diseases on warfarin (Cohort 1) before and after the propensity score matching.**

| **Cohort 1 (N = 8,009) and cohort 2 (N = 344,314) characteristics before propensity score matching** | | | | | | | | | |
| --- | --- | --- | --- | --- | --- | --- | --- | --- | --- |
|  | **Demographics** | | | | | | | | |
|  |  | Cohort | |  | Mean ± SD | Patients | % of Cohort | P-Value | Std diff. |
|  |  | 1 2 | AI | Age at Index | 68.8 +/- 13.1 71.4 +/- 12.3 |  | 100% 100% | <0.001 | 0.204 |
|  |  | 1 2 | 2106-3 | White |  | 5,168 258,281 | 67.8% 77.0% | <0.001 | 0.206 |
|  |  | 1 2 | F | Female |  | 5,323 137,459 | 69.9% 41.0% | <0.001 | 0.607 |
|  |  | 1 2 | 2054-5 | Black or African American |  | 967 22,477 | 12.7% 6.7% | <0.001 | 0.204 |
|  |  | 1 2 | 2028-9 | Asian |  | 422 5,300 | 5.5% 1.6% | <0.001 | 0.215 |
|  | **Diagnosis** | | | | | | | | |
|  |  | Cohort | |  | Mean ± SD | Patients | % of Cohort | P-Value | Std diff. |
|  |  | 1 2 | I50 | Heart failure |  | 2,553 64,372 | 33.5% 19.2% | <0.001 | 0.329 |
|  |  | 1 2 | I20-I25 | Ischemic heart diseases |  | 2,634 75,900 | 34.6% 22.6% | <0.001 | 0.267 |
|  |  | 1 2 | I63 | Cerebral infarction |  | 633 16,993 | 8.3% 5.1% | <0.001 | 0.130 |
|  |  | 1 2 | E08-E13 | Diabetes mellitus |  | 2,073 55,959 | 27.2% 16.7% | <0.001 | 0.256 |
|  |  | 1 2 | E66 | Obesity |  | 1,231 26,130 | 16.2% 7.8% | <0.001 | 0.260 |
|  |  | 1 2 | E78 | Dyslipidemia |  | 3,282 91,334 | 43.1% 27.2% | <0.001 | 0.337 |
|  |  | 1 2 | N18 | Chronic kidney disease |  | 1,930 33,195 | 25.3% 9.9% | <0.001 | 0.414 |
|  |  | 1 2 | I10-I1A | Hypertension |  | 4,915 129,969 | 64.5% 38.7% | <0.001 | 0.534 |
|  | **Medication** | | | | | | | | |
|  |  | Cohort | |  | Mean ± SD | Patients | % of Cohort | P-Value | Std diff. |
|  |  | 1 2 | CV350 | ANTILIPEMIC AGENTS |  | 3,229 122,260 | 42.4% 36.4% | <0.001 | 0.122 |
|  |  | 1 2 | CV700 | DIURETICS |  | 4,112 126,260 | 54.0% 37.6% | <0.001 | 0.332 |
|  |  | 1 2 | CV300 | ANTIARRHYTHMICS |  | 3,073 88,685 | 40.3% 26.4% | <0.001 | 0.298 |
|  |  | 1 2 | CV100 | BETA BLOCKERS/RELATED |  | 4,262 154,516 | 55.9% 46.1% | <0.001 | 0.199 |
|  |  | 1 2 | CV200 | CALCIUM CHANNEL BLOCKERS |  | 2,842 84,527 | 37.3% 25.2% | <0.001 | 0.263 |
|  |  | 1 2 | CV800 | ACE INHIBITORS |  | 2,003 76,217 | 26.3% 22.7% | <0.001 | 0.083 |
|  |  | 1 2 | CV805 | ANGIOTENSIN II RECEPTOR INHIBITOR |  | 1,386 36,155 | 18.2% 10.8% | <0.001 | 0.212 |
|  |  | 1 2 | BL117 | PLATELET AGGREGATION INHIBITORS |  | 3,041 104,355 | 39.9% 31.1% | <0.001 | 0.185 |
| **Cohort 1 (N = 7,611) and cohort 2 (N = 7,611) characteristics after propensity score matching** | | | | | | | | | |
|  | **Demographics** | | | | | | | | |
|  |  | Cohort | |  | Mean ± SD | Patients | % of Cohort | P-Value | Std diff. |
|  |  | 1 2 | AI | Age at Index | 68.9 +/- 13.1 69.0 +/- 13.1 |  | 100% 100% | 0.601 | 0.008 |
|  |  | 1 2 | 2106-3 | White |  | 5,168 5,287 | 67.9% 69.5% | 0.038 | 0.034 |
|  |  | 1 2 | F | Female |  | 5,314 5,313 | 69.8% 69.8% | 0.986 | <0.001 |
|  |  | 1 2 | 2054-5 | Black or African American |  | 967 947 | 12.7% 12.4% | 0.625 | 0.008 |
|  |  | 1 2 | 2028-9 | Asian |  | 413 400 | 5.4% 5.3% | 0.639 | 0.008 |
|  | **Diagnosis** | | | | | | | | |
|  |  | Cohort | |  | Mean ± SD | Patients | % of Cohort | P-Value | Std diff. |
|  |  | 1 2 | I50 | Heart failure |  | 2,547 2,614 | 33.5% 34.3% | 0.251 | 0.019 |
|  |  | 1 2 | I20-I25 | Ischemic heart diseases |  | 2,629 2,719 | 34.5% 35.7% | 0.127 | 0.025 |
|  |  | 1 2 | I63 | Cerebral infarction |  | 633 608 | 8.3% 8.0% | 0.459 | 0.012 |
|  |  | 1 2 | E08-E13 | Diabetes mellitus |  | 2,070 2,087 | 27.2% 27.4% | 0.757 | 0.005 |
|  |  | 1 2 | E66 | Obesity |  | 1,230 1,184 | 16.2% 15.6% | 0.307 | 0.017 |
|  |  | 1 2 | E78 | Dyslipidemia |  | 3,278 3,355 | 43.1% 44.1% | 0.208 | 0.020 |
|  |  | 1 2 | N18 | Chronic kidney disease |  | 1,921 1,875 | 25.2% 24.6% | 0.389 | 0.014 |
|  |  | 1 2 | I10-I1A | Hypertension |  | 4,906 4,973 | 64.5% 65.3% | 0.255 | 0.018 |
|  | **Medication** | | | | | | | | |
|  |  | Cohort | |  | Mean ± SD | Patients | % of Cohort | P-Value | Std diff. |
|  |  | 1 2 | CV350 | ANTILIPEMIC AGENTS |  | 3,227 3,391 | 42.4% 44.6% | 0.007 | 0.043 |
|  |  | 1 2 | CV700 | DIURETICS |  | 4,103 4,236 | 53.9% 55.7% | 0.030 | 0.035 |
|  |  | 1 2 | CV300 | ANTIARRHYTHMICS |  | 3,066 3,085 | 40.3% 40.5% | 0.754 | 0.005 |
|  |  | 1 2 | CV100 | BETA BLOCKERS/RELATED |  | 4,255 4,367 | 55.9% 57.4% | 0.067 | 0.030 |
|  |  | 1 2 | CV200 | CALCIUM CHANNEL BLOCKERS |  | 2,833 2,888 | 37.2% 37.9% | 0.357 | 0.015 |
|  |  | 1 2 | CV800 | ACE INHIBITORS |  | 2,001 1,984 | 26.3% 26.1% | 0.754 | 0.005 |
|  |  | 1 2 | CV805 | ANGIOTENSIN II RECEPTOR INHIBITOR |  | 1,382 1,379 | 18.2% 18.1% | 0.950 | 0.001 |
|  |  | 1 2 | BL117 | PLATELET AGGREGATION INHIBITORS |  | 3,038 3,096 | 39.9% 40.7% | 0.338 | 0.016 |

**Supplementary table 8. Baseline characteristics of patients with atrial fibrillation and autoimmune diseases on non-vitamin K oral anticoagulants (Cohort 1) before and after the propensity score matching.**

| **Cohort 1 (N = 4,801) and cohort 2 (N = 270,241) characteristics before propensity score matching** | | | | | | | | | |
| --- | --- | --- | --- | --- | --- | --- | --- | --- | --- |
|  | **Demographics** | | | | | | | | |
|  |  | Cohort | |  | Mean ± SD | Patients | % of Cohort | P-Value | Std diff. |
|  |  | 1 2 | AI | Age at Index | 70.3 +/- 11.8 70.9 +/- 11.8 |  | 100% 100% | 0.001 | 0.050 |
|  |  | 1 2 | 2106-3 | White |  | 3,347 211,286 | 69.7% 78.2% | <0.001 | 0.194 |
|  |  | 1 2 | F | Female |  | 3,414 110,486 | 71.1% 40.9% | <0.001 | 0.639 |
|  |  | 1 2 | 2054-5 | Black or African American |  | 574 16,965 | 12.0% 6.3% | <0.001 | 0.198 |
|  |  | 1 2 | 2028-9 | Asian |  | 294 5,355 | 6.1% 2.0% | <0.001 | 0.211 |
|  | **Diagnosis** | | | | | | | | |
|  |  | Cohort | |  | Mean ± SD | Patients | % of Cohort | P-Value | Std diff. |
|  |  | 1 2 | I50 | Heart failure |  | 1,460 44,514 | 30.4% 16.5% | <0.001 | 0.334 |
|  |  | 1 2 | I20-I25 | Ischemic heart diseases |  | 1,527 56,904 | 31.8% 21.1% | <0.001 | 0.246 |
|  |  | 1 2 | I63 | Cerebral infarction |  | 418 14,210 | 8.7% 5.3% | <0.001 | 0.136 |
|  |  | 1 2 | E08-E13 | Diabetes mellitus |  | 1,222 43,310 | 25.5% 16.0% | <0.001 | 0.234 |
|  |  | 1 2 | E66 | Obesity |  | 782 25,083 | 16.3% 9.3% | <0.001 | 0.211 |
|  |  | 1 2 | E78 | Dyslipidemia |  | 2,147 83,235 | 44.7% 30.8% | <0.001 | 0.290 |
|  |  | 1 2 | N18 | Chronic kidney disease |  | 940 21,857 | 19.6% 8.1% | <0.001 | 0.338 |
|  |  | 1 2 | I10-I1A | Hypertension |  | 3,117 115,445 | 64.9% 42.7% | <0.001 | 0.457 |
|  | **Medication** | | | | | | | | |
|  |  | Cohort | |  | Mean ± SD | Patients | % of Cohort | P-Value | Std diff. |
|  |  | 1 2 | CV350 | ANTILIPEMIC AGENTS |  | 2,072 95,481 | 43.2% 35.3% | <0.001 | 0.161 |
|  |  | 1 2 | CV700 | DIURETICS |  | 2,351 86,111 | 49.0% 31.9% | <0.001 | 0.354 |
|  |  | 1 2 | CV300 | ANTIARRHYTHMICS |  | 2,187 75,750 | 45.6% 28.0% | <0.001 | 0.370 |
|  |  | 1 2 | CV100 | BETA BLOCKERS/RELATED |  | 2,791 124,278 | 58.1% 46.0% | <0.001 | 0.245 |
|  |  | 1 2 | CV200 | CALCIUM CHANNEL BLOCKERS |  | 1,930 71,520 | 40.2% 26.5% | <0.001 | 0.295 |
|  |  | 1 2 | CV800 | ACE INHIBITORS |  | 1,043 53,217 | 21.7% 19.7% | <0.001 | 0.050 |
|  |  | 1 2 | CV805 | ANGIOTENSIN II RECEPTOR INHIBITOR |  | 979 34,959 | 20.4% 12.9% | <0.001 | 0.201 |
|  |  | 1 2 | BL117 | PLATELET AGGREGATION INHIBITORS |  | 1,851 77,627 | 38.6% 28.7% | <0.001 | 0.209 |
| **Cohort 1 (N = 4,800) and cohort 2 (N = 4,800) characteristics after propensity score matching** | | | | | | | | | |
|  | **Demographics** | | | | | | | | |
|  |  | Cohort | |  | Mean ± SD | Patients | % of Cohort | P-Value | Std diff. |
|  |  | 1 2 | AI | Age at Index | 70.3 +/- 11.8 70.5 +/- 12.1 |  | 100% 100% | 0.360 | 0.019 |
|  |  | 1 2 | 2106-3 | White |  | 3,347 3,417 | 69.7% 71.2% | 0.117 | 0.032 |
|  |  | 1 2 | F | Female |  | 3,413 3,435 | 71.1% 71.6% | 0.620 | 0.010 |
|  |  | 1 2 | 2054-5 | Black or African American |  | 574 553 | 12.0% 11.5% | 0.506 | 0.014 |
|  |  | 1 2 | 2028-9 | Asian |  | 293 275 | 6.1% 5.7% | 0.436 | 0.016 |
|  | **Diagnosis** | | | | | | | | |
|  |  | Cohort | |  | Mean ± SD | Patients | % of Cohort | P-Value | Std diff. |
|  |  | 1 2 | I50 | Heart failure |  | 1,459 1,484 | 30.4% 30.9% | 0.580 | 0.011 |
|  |  | 1 2 | I20-I25 | Ischemic heart diseases |  | 1,526 1,546 | 31.8% 32.2% | 0.662 | 0.009 |
|  |  | 1 2 | I63 | Cerebral infarction |  | 417 386 | 8.7% 8.0% | 0.253 | 0.023 |
|  |  | 1 2 | E08-E13 | Diabetes mellitus |  | 1,222 1,204 | 25.5% 25.1% | 0.672 | 0.009 |
|  |  | 1 2 | E66 | Obesity |  | 782 759 | 16.3% 15.8% | 0.523 | 0.013 |
|  |  | 1 2 | E78 | Dyslipidemia |  | 2,147 2,134 | 44.7% 44.5% | 0.790 | 0.005 |
|  |  | 1 2 | N18 | Chronic kidney disease |  | 939 913 | 19.6% 19.0% | 0.501 | 0.014 |
|  |  | 1 2 | I10-I1A | Hypertension |  | 3,116 3,131 | 64.9% 65.2% | 0.748 | 0.007 |
|  | **Medication** | | | | | | | | |
|  |  | Cohort | |  | Mean ± SD | Patients | % of Cohort | P-Value | Std diff. |
|  |  | 1 2 | CV350 | ANTILIPEMIC AGENTS |  | 2,072 2,092 | 43.2% 43.6% | 0.680 | 0.008 |
|  |  | 1 2 | CV700 | DIURETICS |  | 2,350 2,413 | 49.0% 50.3% | 0.198 | 0.026 |
|  |  | 1 2 | CV300 | ANTIARRHYTHMICS |  | 2,187 2,214 | 45.6% 46.1% | 0.580 | 0.011 |
|  |  | 1 2 | CV100 | BETA BLOCKERS/RELATED |  | 2,790 2,892 | 58.1% 60.3% | 0.034 | 0.043 |
|  |  | 1 2 | CV200 | CALCIUM CHANNEL BLOCKERS |  | 1,929 1,988 | 40.2% 41.4% | 0.220 | 0.025 |
|  |  | 1 2 | CV800 | ACE INHIBITORS |  | 1,043 1,069 | 21.7% 22.3% | 0.522 | 0.013 |
|  |  | 1 2 | CV805 | ANGIOTENSIN II RECEPTOR INHIBITOR |  | 978 920 | 20.4% 19.2% | 0.137 | 0.030 |
|  |  | 1 2 | BL117 | PLATELET AGGREGATION INHIBITORS |  | 1,851 1,840 | 38.6% 38.3% | 0.817 | 0.005 |

**Supplementary table 8. Baseline characteristics of patients with atrial fibrillation and autoimmune diseases on non-vitamin K oral anticoagulants (Cohort 1) or on non-vitamin K antagonist oral anticoagulant (Cohort 2) before and after the propensity score matching.**

| **Cohort 1 (N = 8,009) and cohort 2 (N = 4,801) characteristics before propensity score matching** | | | | | | | | | |
| --- | --- | --- | --- | --- | --- | --- | --- | --- | --- |
|  | **Demographics** | | | | | | | | |
|  |  | Cohort | |  | Mean ± SD | Patients | % of Cohort | P-Value | Std diff. |
|  |  | 1 2 | AI | Age at Index | 68.8 +/- 13.1 70.3 +/- 11.8 |  | 100% 100% | <0.001 | 0.116 |
|  |  | 1 2 | 2106-3 | White |  | 5,168 3,347 | 67.8% 69.7% | 0.027 | 0.041 |
|  |  | 1 2 | F | Female |  | 5,323 3,414 | 69.9% 71.1% | 0.136 | 0.028 |
|  |  | 1 2 | 2054-5 | Black or African American |  | 967 574 | 12.7% 12.0% | 0.227 | 0.022 |
|  |  | 1 2 | 2028-9 | Asian |  | 422 294 | 5.5% 6.1% | 0.173 | 0.025 |
|  | **Diagnosis** | | | | | | | | |
|  |  | Cohort | |  | Mean ± SD | Patients | % of Cohort | P-Value | Std diff. |
|  |  | 1 2 | I50 | Heart failure |  | 2,553 1,460 | 33.5% 30.4% | <0.001 | 0.066 |
|  |  | 1 2 | I20-I25 | Ischemic heart diseases |  | 2,634 1,527 | 34.6% 31.8% | 0.001 | 0.059 |
|  |  | 1 2 | I63 | Cerebral infarction |  | 633 418 | 8.3% 8.7% | 0.436 | 0.014 |
|  |  | 1 2 | E08-E13 | Diabetes mellitus |  | 2,073 1,222 | 27.2% 25.5% | 0.031 | 0.040 |
|  |  | 1 2 | E66 | Obesity |  | 1,231 782 | 16.2% 16.3% | 0.844 | 0.004 |
|  |  | 1 2 | E78 | Dyslipidemia |  | 3,282 2,147 | 43.1% 44.7% | 0.071 | 0.033 |
|  |  | 1 2 | N18 | Chronic kidney disease |  | 1,930 940 | 25.3% 19.6% | <0.001 | 0.138 |
|  |  | 1 2 | I10-I1A | Hypertension |  | 4,915 3,117 | 64.5% 64.9% | 0.631 | 0.009 |
|  | **Medication** | | | | | | | | |
|  |  | Cohort | |  | Mean ± SD | Patients | % of Cohort | P-Value | Std diff. |
|  |  | 1 2 | CV350 | ANTILIPEMIC AGENTS |  | 3,229 2,072 | 42.4% 43.2% | 0.391 | 0.016 |
|  |  | 1 2 | CV700 | DIURETICS |  | 4,112 2,351 | 54.0% 49.0% | <0.001 | 0.100 |
|  |  | 1 2 | CV300 | ANTIARRHYTHMICS |  | 3,073 2,187 | 40.3% 45.6% | <0.001 | 0.106 |
|  |  | 1 2 | CV100 | BETA BLOCKERS/RELATED |  | 4,262 2,791 | 55.9% 58.1% | 0.016 | 0.044 |
|  |  | 1 2 | CV200 | CALCIUM CHANNEL BLOCKERS |  | 2,842 1,930 | 37.3% 40.2% | 0.001 | 0.060 |
|  |  | 1 2 | CV800 | ACE INHIBITORS |  | 2,003 1,043 | 26.3% 21.7% | <0.001 | 0.107 |
|  |  | 1 2 | CV805 | ANGIOTENSIN II RECEPTOR INHIBITOR |  | 1,386 979 | 18.2% 20.4% | 0.002 | 0.056 |
|  |  | 1 2 | BL117 | PLATELET AGGREGATION INHIBITORS |  | 3,041 1,851 | 39.9% 38.6% | 0.133 | 0.028 |
| **Cohort 1 (N = 4,733) and cohort 2 (N = 4,733) characteristics after propensity score matching** | | | | | | | | | |
|  | **Demographics** | | | | | | | | |
|  |  | Cohort | |  | Mean ± SD | Patients | % of Cohort | P-Value | Std diff. |
|  |  | 1 2 | AI | Age at Index | 70.2 +/- 12.4 70.2 +/- 11.8 |  | 100% 100% | 0.931 | 0.002 |
|  |  | 1 2 | 2106-3 | White |  | 3,308 3,303 | 69.9% 69.8% | 0.911 | 0.002 |
|  |  | 1 2 | F | Female |  | 3,355 3,363 | 70.9% 71.1% | 0.856 | 0.004 |
|  |  | 1 2 | 2054-5 | Black or African American |  | 572 568 | 12.1% 12.0% | 0.899 | 0.003 |
|  |  | 1 2 | 2028-9 | Asian |  | 271 282 | 5.7% 6.0% | 0.630 | 0.010 |
|  | **Diagnosis** | | | | | | | | |
|  |  | Cohort | |  | Mean ± SD | Patients | % of Cohort | P-Value | Std diff. |
|  |  | 1 2 | I50 | Heart failure |  | 1,445 1,448 | 30.5% 30.6% | 0.947 | 0.001 |
|  |  | 1 2 | I20-I25 | Ischemic heart diseases |  | 1,522 1,513 | 32.2% 32.0% | 0.843 | 0.004 |
|  |  | 1 2 | I63 | Cerebral infarction |  | 413 410 | 8.7% 8.7% | 0.913 | 0.002 |
|  |  | 1 2 | E08-E13 | Diabetes mellitus |  | 1,199 1,204 | 25.3% 25.4% | 0.906 | 0.002 |
|  |  | 1 2 | E66 | Obesity |  | 764 768 | 16.1% 16.2% | 0.911 | 0.002 |
|  |  | 1 2 | E78 | Dyslipidemia |  | 2,083 2,104 | 44.0% 44.5% | 0.664 | 0.009 |
|  |  | 1 2 | N18 | Chronic kidney disease |  | 950 939 | 20.1% 19.8% | 0.777 | 0.006 |
|  |  | 1 2 | I10-I1A | Hypertension |  | 3,068 3,057 | 64.8% 64.6% | 0.813 | 0.005 |
|  | **Medication** | | | | | | | | |
|  |  | Cohort | |  | Mean ± SD | Patients | % of Cohort | P-Value | Std diff. |
|  |  | 1 2 | CV350 | ANTILIPEMIC AGENTS |  | 2,051 2,036 | 43.3% 43.0% | 0.756 | 0.006 |
|  |  | 1 2 | CV700 | DIURETICS |  | 2,341 2,339 | 49.5% 49.4% | 0.967 | 0.001 |
|  |  | 1 2 | CV300 | ANTIARRHYTHMICS |  | 2,147 2,123 | 45.4% 44.9% | 0.620 | 0.010 |
|  |  | 1 2 | CV100 | BETA BLOCKERS/RELATED |  | 2,725 2,734 | 57.6% 57.8% | 0.851 | 0.004 |
|  |  | 1 2 | CV200 | CALCIUM CHANNEL BLOCKERS |  | 1,888 1,883 | 39.9% 39.8% | 0.916 | 0.002 |
|  |  | 1 2 | CV800 | ACE INHIBITORS |  | 1,031 1,041 | 21.8% 22.0% | 0.804 | 0.005 |
|  |  | 1 2 | CV805 | ANGIOTENSIN II RECEPTOR INHIBITOR |  | 964 944 | 20.4% 19.9% | 0.608 | 0.011 |
|  |  | 1 2 | BL117 | PLATELET AGGREGATION INHIBITORS |  | 1,861 1,833 | 39.3% 38.7% | 0.555 | 0.012 |
